# Aporofobia na atenção primária à saúde: percepção de profissionais da saúde sobre preconceito contra os pobres

**DOI:** 10.1590/0102-311XPT149023

**Published:** 2026-01-09

**Authors:** Brena Barreto Barbosa, Jessica Freire dos Santos Veras, Lia Silveira Adriano, Raquel Canuto, Antonio Augusto Ferreira Carioca

**Affiliations:** 1 Universidade de Fortaleza, Fortaleza, Brasil.; 2 Universidade Federal do Rio Grande do Sul, Porto Alegre, Brasil.

**Keywords:** Profissionais de Saúde, Pobreza, Preconceito, Estratégia Saúde da Família, Health Personnel, Poverty, Prejudice, Family Health Strategy, Personal de Salud, Pobreza, Prejuicio, Estrategia de Salud Familiar

## Abstract

A aporofobia compromete o acesso e a qualidade do cuidado nos serviços de saúde. Nesse contexto, os profissionais da Estratégia Saúde da Família (ESF) exercem papel central para uma assistência à saúde universal, integral e equânime. O objetivo deste estudo foi compreender percepções sobre a aporofobia entre profissionais de nível superior da ESF. Trata-se de estudo de abordagem mista sequencial explanatória, realizado com 96 profissionais da ESF em Fortaleza, Ceará, Brasil. Na primeira etapa, foram coletados dados quantitativos, a partir do *Questionário de Atitudes em Relação à População em Situação de Sem-Abrigo*. Na fase qualitativa, 10 profissionais foram entrevistados conforme pontuação no questionário (menor quintil e maior quintil). A análise de dados utilizou o software IraMuTeq, incluindo análise de similitude, e interpretada à luz das teorias da filósofa Adela Cortina. Os resultados indicaram associação significativa entre aporofobia e cor da pele (p = 0,022) e vínculo empregatício (p = 0,008). O conteúdo qualitativo foi categorizado em quatro classes: causas relacionadas à pobreza; manifestações de aporofobia e seus estereótipos; integralidade no atendimento a grupos vulneráveis; e humanização e adaptação no atendimento. As percepções dos profissionais variaram entre práticas humanizadas e preconceitos sutis ou explícitos, evidenciando desafios na garantia da equidade no cuidado. Esses resultados reforçam a necessidade de capacitação contínua dos profissionais da ESF, da inclusão de discussões sobre determinação social da saúde na formação acadêmica e do fortalecimento da intersetorialidade para garantir um cuidado mais humanizado e inclusivo.

## Abstract (en)

Aporophobia compromises access to and the quality of care at health services. Health professionals of the Family Health Strategy (FHS) play a central role in ensuring universal, comprehensive, equitable health care. The aim of the present study was to understand perceptions about aporophobia among higher-level FHS professionals. A sequential, explanatory, mixed-methods study was conducted with 96 FHS professionals in Fortaleza, Ceará State, Brazil. In the first stage, quantitative data were collected using the *Attitudes Towards the Homeless Questionnaire*. In the qualitative phase, 10 professionals were interviewed based on the scores they received on the questionnaire (lowest and highest quintiles). The data were analyzed using the IraMuTeq software and included similarity analysis interpreted in light of the theories of philosopher Adela Cortina. Significant associations were found between aporophobia and both skin color (p = 0.022) and employment status (p = 0.008). The qualitative content was categorized into four classes: poverty-related causes; manifestations of aporophobia and its stereotypes; comprehensive care for vulnerable groups; and humanization and adaptation in care. The perceptions of the professionals ranged from humanized practices to subtle or even explicit prejudices, revealing challenges in the assurance of equity in care. These results underscore the need for ongoing training of healthcare providers of the FHS, the inclusion of discussions on the social determinants of health at higher education institutions, and the strengthening of intersectoral collaboration to ensure more humanized and inclusive care.

## Abstract (es)

La aporofobia compromete el acceso y la calidad de la atención en los servicios de salud. En este contexto, los profesionales de la Estrategia Salud de Familiar (ESF) desempeñan un papel central en la atención médica universal, integral y equitativa. El objetivo de este estudio fue identificar las percepciones de los profesionales de la educación superior de ESF acerca de la aporofobia. Se trata de un estudio explicativo de abordaje mixto secuencial realizado con 96 profesionales de ESF en Fortaleza, Ceará (Brasil). En la primera etapa, se recopilaron datos cuantitativos del *Cuestionario de Actitudes con Relación a la Población sin Hogar*. En la fase cualitativa se entrevistó a 10 profesionales según la puntuación del Cuestionario (quintil más bajo y quintil más alto). El análisis de datos utilizó el software IraMuTeq, incluido el análisis de similitud, interpretado a la luz de las teorías de la filósofa Adela Cortina. Los resultados indicaron una asociación significativa entre la aforofobia y color de la piel (p = 0,022) y situación laboral (p = 0,008). El contenido cualitativo se clasificó en cuatro clases: causas relacionadas con la pobreza; manifestaciones de la aporofobia y sus estereotipos; integralidad en la atención a grupos vulnerables; y humanización y adaptación en la atención. Las percepciones de los profesionales oscilaban entre prácticas humanizadas y prejuicios sutiles o explícitos, con énfasis en los desafíos para garantizar la equidad en la atención. Estos resultados refuerzan la necesidad de una formación continua de los profesionales de la ESF, de la inclusión de discusiones sobre la determinación social de la salud en la formación académica y del fortalecimiento de la intersectorialidad para garantizar una atención más humanizada e inclusiva.

## Introdução

A aporofobia, definida como aversão, discriminação e preconceito contra os pobres, é um fenômeno que afeta diversos aspectos da vida social, incluindo o acesso e a qualidade dos serviços de saúde ^1^. Essa discriminação pode se manifestar de diferentes formas, desde atitudes sutis até comportamentos explícitos que comprometem a dignidade e o bem-estar dos indivíduos em situação de vulnerabilidade econômica ^2^. No contexto da saúde pública, a aporofobia pode resultar em um atendimento desigual, reforçando as barreiras já existentes para as populações socioeconomicamente desfavorecidas ^3^.

Esse conceito, popularizado pela filósofa espanhola Adela Cortina ^1^, foi inicialmente utilizado para descrever a aversão direcionada a pessoas pobres no contexto europeu, com destaque para imigrantes em situação de vulnerabilidade. No entanto, no Brasil, a aporofobia é atravessada pelo racismo, uma vez que a formação social, econômica e política do país é nele baseada. Assim, renda e raça se relacionam, de modo que a aversão ao pobre muitas vezes é também a aversão ao negro e ao indígena no Brasil ^4^. Autores como Gonzalez ^5^, Nascimento ^6^ e Fanon ^7^ apontam que a marginalização de minorias raciais é resultado de um projeto histórico que legitima a exclusão e está presente de forma estrutural, influenciando, inclusive, as práticas de cuidado na saúde. Nesse contexto, pode-se compreender como essas estruturas de poder determinam quais grupos têm acesso a condições dignas de cuidado e quais permanecem expostos a situações de adoecimento e morte evitáveis ^8^.

A Estratégia Saúde da Família (ESF) é uma política pública prioritária no Brasil, voltada à expansão da atenção básica, em conformidade com os princípios do Sistema Único de Saúde (SUS). Seu objetivo é promover saúde e prevenir doenças por meio de ações comunitárias e fortalecimento da atenção primária à saúde (APS) ^9^. A maioria da população brasileira depende do SUS, que garante acesso à saúde para as parcelas mais vulneráveis, promovendo equidade ^10^. Os profissionais da ESF têm um papel central, oferecendo atendimento universal, integral e equânime, assegurando cuidados de qualidade para todos os usuários da atenção básica ^11^.

A discriminação nos serviços de saúde é um desafio que compromete a equidade no atendimento, afetando especialmente grupos vulneráveis ^12^. Dados da *Pesquisa Nacional de Saúde* (PNS) de 2013 revelam que mais de 10% dos adultos sofreram discriminação, associada, sobretudo, à pobreza e à classe social, atingindo principalmente mulheres, pessoas com baixa escolaridade, negras e sem plano privado ^13^. Em estudo em Porto Alegre (Rio Grande do Sul) e Florianópolis (Santa Catarina), a prevalência foi semelhante, mais frequente entre negros, fumantes e indivíduos de 31 a 40 anos, tendo como causa central a posição socioeconômica ^14^. Para a população em situação de rua, o estigma da pobreza intensifica a exclusão, gerando desassistência e negligência ^15^.

A incapacidade de lidar com a diversidade, julgamentos prejudiciais e atitudes preconceituosas são alguns dos fatores que comprometem a comunicação efetiva entre profissionais de saúde e usuários. Esses elementos impactam negativamente a satisfação e utilização dos serviços de saúde, além de afetar o cuidado em saúde ^16^. Nesse sentido, é fundamental que os profissionais da saúde adotem postura inclusiva para garantir um atendimento equitativo e de qualidade a todos os usuários.

Não foram identificados estudos no Brasil que abordem a percepção de profissionais da ESF sobre a aporofobia. Ao investigar como esses profissionais entendem e vivenciam o preconceito contra a pobreza em seu cotidiano de trabalho, espera-se contribuir para a construção de um sistema de saúde que atenda com equidade todas as camadas da população. Portanto, este estudo teve como objetivo compreender percepções sobre aporofobia entre profissionais de nível superior da ESF.

## Métodos

### Delineamento do estudo

Trata-se de estudo de abordagem mista sequencial explanatória, realizado com profissionais de nível superior vinculados à ESF, na cidade de Fortaleza, capital do Ceará, Brasil, entre janeiro e abril de 2024, envolvendo duas fases distintas. Na primeira etapa, foram coletados dados quantitativos, com base em um estudo exploratório, que orientaram a seleção intencional dos participantes para a segunda fase, de caráter qualitativo (estudo de caso único e descritivo) ^17^. A pesquisa foi aprovada no Comitê de Ética em Pesquisa da Universidade de Fortaleza (parecer nº 6.552.992).

### Cenário

A cidade de Fortaleza possui uma população de 2,4 milhões de habitantes ^18^. O município conta com 123 unidades básicas de saúde (UBS) e 472 equipes da ESF distribuídas em seus 121 bairros, todos situados na área urbana e agrupados em seis regiões administrativas. Essas regiões são geridas pelas Secretarias Executivas Regionais (SER), que reúnem bairros com proximidade geográfica e características socioeconômicas semelhantes ^19^.

### Fase 1 - quantitativa

Os participantes da fase quantitativa foram profissionais de saúde de nível superior da ESF, atuantes em UBS de Fortaleza, ativos na rede pública durante a coleta. Excluíram-se profissionais que não atuam diretamente com usuários, realizam atendimentos esporádicos ou que estavam afastados no período de coleta.

A seleção das UBS participantes ocorreu por sorteio entre regiões administrativas. Em cada UBS, foi incluída ao menos uma equipe da ESF, garantindo representatividade. O critério adotado buscou contemplar três UBS em cada uma das seis regionais de Fortaleza. Os profissionais de nível superior da ESF foram convidados em seus locais de trabalho, visando compor uma amostra intencional de 16 participantes por SER. Assim, inicialmente, participaram 96 profissionais de saúde, que atenderam aos critérios de inclusão e exclusão, compondo a base para a realização do estudo.

Para coleta das informações quantitativas foi aplicado questionário estruturado sobre informações demográficas, socioeconômicas e educacionais. As variáveis avaliadas no questionário incluíram: faixa etária (< 40 anos ou ≥ 40 anos); sexo (masculino ou feminino); e categoria profissional, (médico, enfermeiro ou dentista); cor da pele autodeclarada (branca, preta ou parda); tipo de instituição de ensino universitário frequentada (instituição pública ou privada); vínculo empregatício (servidor público estatutário, celetista ou temporário); e renda familiar mensal (de 2 a 6 salários mínimos, ou 7, ou mais salários). As variáveis foram descritas em valores absolutos (n) e percentuais (%).

Para medir os níveis de aporofobia entre os profissionais em relação aos usuários dos serviços públicos de saúde, foi utilizado o *Questionário de Atitudes em Relação à População em Situação de Sem-Abrigo* (QARPSSA) ^20^. Originalmente desenvolvido em Portugal para avaliar atitudes, percepções e práticas em relação a pessoas em situação de sem-abrigo, esse questionário é composto por 32 itens, divididos em três dimensões: cognitiva, emocional e comportamental.

Para cada item nas três dimensões, os participantes são solicitados a indicar o grau de concordância com as afirmações em uma escala de cinco pontos, que vai de “discordo totalmente” (1) a “concordo totalmente” (5). O questionário apresenta afirmações em polos distintos (positivo e negativo), permitindo uma análise da amplitude de atitudes. Pontuações mais altas representam atitudes mais positivas ^20^.

Embora a aporofobia seja um conceito recente e ainda não existam instrumentos específicos para sua avaliação, o QARPSSA foi utilizado neste estudo como uma *proxy* adequada aos objetivos propostos. Reconhece-se, contudo, que o questionário não foi originalmente desenvolvido para medir aporofobia de forma direta e foi elaborado em um contexto sociocultural distinto do brasileiro, o que pode influenciar a interpretação das afirmações pelos participantes. Sua aplicação ocorreu em caráter exploratório, visando demonstrar o potencial do instrumento no campo da saúde pública. Ressalta-se, entretanto, a importância de aprofundar pesquisas que esclareçam em que medida atitudes em relação à população em situação de sem-abrigo se relacionam à aporofobia no Brasil. Diante da inexistência, até o momento, de instrumentos validados para mensuração específica desse construto, justificou-se a adoção do QARPSSA como alternativa metodológica para a análise.

Ainda assim, sua estrutura permite avaliar os estereótipos e as atitudes negativas em relação a grupos socialmente marginalizados. Isso se deve ao fato de que o preconceito e a discriminação contra pessoas em situação de pobreza estão relacionados com as atitudes e percepções em relação a indivíduos em situação de vulnerabilidade, como as pessoas sem-abrigo, conforme descrito por Cortina ^1^.

### Fase 2 - qualitativa

Os 96 participantes do questionário quantitativo tiveram suas pontuações no QARPSSA avaliadas e divididas em quintis. Dentre eles, 19 foram classificados no menor quintil e 19 no maior quintil. Esses 38 participantes dos quintis extremos foram contactados de modo aleatório; dez aceitaram participar da fase qualitativa, sendo seis do menor quintil e quatro do maior quintil. Esses grupos representam, respectivamente, os profissionais com menor preconceito ao lidar com o público em situação de vulnerabilidade econômica e aqueles que manifestaram comportamentos mais próximos de preconceito, discriminação e aversão aos usuários em situação de pobreza.

Foram realizadas entrevistas semi-estruturadas por uma pesquisadora (estudante de pós-graduação em Saúde Coletiva) que não conhecia a informação sobre a classificação prévia do QARPSSA. As entrevistas foram conduzidas com base em roteiro semiestruturado com as seguintes perguntas: (1) O que é pobreza? E quais suas causas?; (2) ⁠Como você conceitua a aversão ao pobre?; (3) Você já presenciou episódios de aversão aos pobres - violência, preconceito, discriminação - na UBS? Conte o caso; (4) Quais foram as suas estratégias de enfrentamento aos episódios citados acima?; (5) ⁠Como você caracteriza o ambiente da UBS para as pessoas pobres? e (6) Como sua formação acadêmica universitária contribui para isso?

As entrevistas ocorreram em locais reservados nas UBS, evitando deslocamentos, e foram agendadas conforme a disponibilidade dos profissionais. As entrevistas foram gravadas em áudio, com duração média de 20 minutos. Para preservar o anonimato, os entrevistados foram identificados pela letra “E” seguida de numeração crescente (E1, E2, E3).

A análise dos dados foi realizada inicialmente com a caracterização dos entrevistados. Os depoimentos foram transcritos, constituindo o *corpus* textual. Cada texto foi identificado com uma linha de comando ordenada de “participante_01” a “participante_10”, com as perguntas suprimidas para focar apenas nos relatos. O arquivo foi salvo no formato UTF-8 (*Unicode Transformation Format 8 bit codeunits*), possibilitando a análise pelo software IraMuTeq (http://www.iramuteq.org).

Os dados foram analisados utilizando o software IraMuTeq. Realizaram-se análises lexicográficas clássicas para compreender os dados estatísticos e quantificar as evocações e formas. A classificação hierárquica descendente (CHD) foi obtida para aferir os dados do dendrograma em função das classes geradas, considerando as palavras com χ^2^ > 3,84 (p < 0,05). A análise de similitude, baseada na teoria dos grafos, foi realizada para identificar as ocorrências e conexões entre as palavras. Por fim, executou-se a análise fatorial por correspondência (AFC).

A interpretação e análise das informações obtidas foram realizadas à luz das teorias da filósofa Adela Cortina ^1^. Outras referências disponíveis na literatura também foram utilizadas para fornecer suporte adequado às inferências sobre os contextos analisados, como as obras de Gonzalez ^5^, Nascimento ^6^ e Fanon ^7^.

## Resultados

Participaram 96 profissionais, a maioria era do sexo feminino (66,7%) e com menos de 40 anos (55,2%). A distribuição entre médicos, enfermeiros e dentistas foi equilibrada, com maior representação de enfermeiros (39,6%). Quanto à cor da pele autodeclarada, a maioria se identificou como parda (52,1%), seguida por branca (40,6%). A maioria dos profissionais cursou ensino universitário em instituições públicas (57,3%), possuía vínculo empregatício como servidor público estatutário (38,5%) e declarou ganhar 7 ou mais salários mínimos (75%).

A Tabela 1 apresenta as características demográficas, socioeconômicas e educacionais dos profissionais da ESF, distribuídas em quintis de aporofobia segundo o escore do QARPSSA. Observou-se significância estatística em relação à cor da pele, com profissionais autodeclarados pretos ausentes nos quintis superiores de aporofobia (Q3, Q4 e Q5) (p = 0,022). O vínculo empregatício também apresentou significância, com maior concentração de servidores estatutários no quintil de menor aporofobia (Q1, 63,2%) e uma predominância de servidores temporários no quintil de maior aporofobia (Q5, 57,9%) (p = 0,008). As demais variáveis avaliadas não apresentaram diferenças significativas entre os quintis.

**Tabela 1:** Variáveis demográficas, socioeconômicas e educacionais de profissionais da saúde da Estratégia Saúde da Família.

Variáveis	QARPSSA (%)					Valor de p *
	Q1 (n = 19)	Q2 (n = 19)	Q3 (n = 20)	Q4 (n = 19)	Q5 (n = 19)	
Faixa etária (anos)						0,135
< 40	31,6	68,4	55,0	52,6	68,4	
≥ 40	68,4	31,6	45,0	47,4	31,6	
Sexo						0,103
Masculino	47,4	15,8	20,0	47,4	36,8	
Feminino	52,6	84,2	80,0	52,6	63,2	
Categoria profissional						0,616
Médico	31,6	21,1	45,0	42,1	52,6	
Enfermeiro	36,8	57,9	35,0	36,8	31,6	
Dentista	31,6	21,1	20,0	21,1	15,8	
Cor da pele						0,022
Branca	52,6	21,1	30,0	47,4	52,6	
Preta	15,8	21,1	0,0	0,0	0,0	
Parda	31,6	57,9	70,0	52,6	47,4	
Tipo de ensino universitário						0,994
Público	52,6	57,9	60,0	57,9	57,9	
Privado	47,4	42,1	40,0	42,1	42,1	
Vínculo empregatício						0,008
Servidor público (estatutário)	63,2	26,3	40,0	47,4	15,8	
Servidor público (celetista)	10,5	57,9	25,0	21,1	26,3	
Servidor temporário	26,3	15,8	35,0	31,6	57,9	
Renda familiar mensal (salários mínimos)						0,359
2-6	21,1	42,1	15,0	21,1	26,3	
≥ 7	78,9	57,9	85,0	78,9	73,7	

Q: quintil; QARPSSA: *Questionário de Atitudes em Relação à População em Situação de Sem-Abrigo*.

* Teste qui-quadrado. Considerou-se significante p < 0,05.

No componente qualitativo, participaram dez profissionais, sendo quatro do quintil alto da pontuação do QARPSSA e seis do quintil baixo da pontuação do QARPSSA.

O *corpus* geral consistiu em dez entrevistas, totalizando 338 segmentos de texto (ST), dos quais 313 (92,60%) foram validados. Identificaram-se 11.314 ocorrências, sendo 1.709 palavras distintas, das quais 887 apareceram apenas uma vez. O conteúdo foi categorizado em quatro classes: 1 - “Causas relacionadas à pobreza” (16,61%); 2 - “Manifestações de aporofobia e seus estereótipos” (32,91%); 3 - “Integralidade no atendimento a grupos vulneráveis” (19,81%); 4 - “Humanização e adaptação no atendimento” (30,67%).

**Figura 1:**
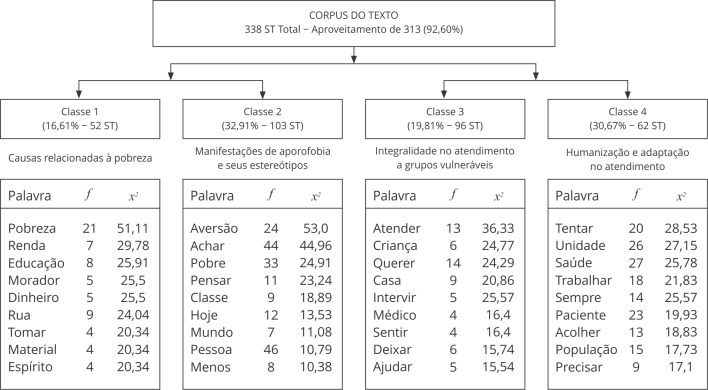
Diagrama de classes.

Com o objetivo de ilustrar melhor as palavras do *corpus* textual em suas respectivas classes, organizou-se um diagrama de classes com exemplos de palavras de cada classe, avaliadas por meio do teste qui-quadrado (χ^2^). Nesse diagrama, emergem as evocações que apresentam vocabulário semelhante entre si e distinto das outras classes. Em seguida, cada uma dessas classes será apresentada, operacionalizada e exemplificada, conforme identificado pela análise de CHD (Figura 1).

ST: segmento de texto.

### Classe 1 - Causas relacionadas à pobreza

Compreende 16,61% (*f* = 52 ST) do *corpus* total analisado. Constituída por palavras e radicais no intervalo entre χ^2^ = 3,86 (Vida) e χ^2^ = 51,11 (Pobreza). Essa classe é composta por palavras como “Pobreza” (χ^2^ = 51,11); “Renda” (χ^2^ = 29,78); “Educação” (χ^2^ = 25,91); “Morador” (χ^2^ = 25,5); “Dinheiro” (χ^2^ = 25,5); “Rua” (χ^2^ = 24,04); “Tomar” (χ^2^ =20,34); “Material” (χ^2^ = 20,34) e “Espírito” (χ^2^ = 20,34).

As falas revelam percepções variadas sobre as causas relacionadas à pobreza. Para alguns, a pobreza é definida pela falta de recursos financeiros, enquanto outros ampliam esse conceito para incluir uma pobreza de espírito, caracterizada por uma vida excessivamente materialista. Há também a visão de que a pobreza está relacionada à falta de educação dos indivíduos perante os profissionais de saúde. Houve associação entre moradores de rua e pobreza, como a ideia de que essas pessoas são vetores de doenças ou potenciais ameaças.

“*Então, pra mim, pobreza no sentido material é aquela pessoa que tem poucos recursos financeiros, certo? Agora, no sentido... Espiritismo, espiritual, lá vai um conceito um pouco mais amplo, porque às vezes a pessoa tem dinheiro, mas ela é pobre de espírito*” (E10 - menor quintil de QARPSSA).

“*Morador de rua, a gente já tá vendo ali que aquela pessoa é pobre. Então, aversão no sentido de achar que vai pegar alguma doença da pessoa, achar que aquela pessoa vai lhe violentar de alguma forma*” (E5 - maior quintil de QARPSSA).

### Classe 2 - Manifestações de aporofobia e seus estereótipos

Compreende 32,91% (*f* = 103 ST) do *corpus* total analisado. Constituída por palavras e radicais no intervalo entre χ^2^ = 3,9 (Social) e χ^2^ = 53,0 (Aversão). Essa classe é composta por palavras como “Aversão” (χ^2^ = 53,0); “Achar” (χ^2^ = 44,96); “Pobre” (χ^2^ = 24,91); “Pensar” (χ^2^ = 23,24); “Classe” (χ^2^ = 18,89); “Hoje” (χ^2^ = 13,53); “Mundo” (χ^2^ = 11,08); “Pessoa” (χ^2^ = 10,79) e “Menos” (χ^2^ = 10,38). 

A análise das falas revelou manifestações de aporofobia e estereótipos, com a pobreza estigmatizada pela aparência e situação de vida. Relatos indicam resistência inicial dos profissionais em atender usuários do SUS e lidar com vulnerabilidades. Além disso, há uma percepção de que a aversão à pobreza também se manifesta em gestos sutis, como o afastamento e o olhar de desprezo.

“*Aversão ao pobre? Assim, infelizmente a gente tem aqueles preconceitos ali, os tabus pré-estabelecidos pela sociedade, né? De que o pobre é aquele da periferia, que geralmente é uma família que, é, muitas vezes depende muito de Bolsa Família*” (E2 - maior quintil de QARPSSA).

“*...na época da faculdade, né?* (...) *eu era da Medicina, mas tinha, tinha é, algumas pessoas, acho que depois melhoraram, né? Mas no começo elas não queriam atender as pessoas do SUS, né?*” (E3 - maior quintil de QARPSSA).

“...*eu acho que a aversão é uma palavra forte.* (...) *Então, a aversão, apesar que tem muita gente que tem, que a gente vê por olhar, por se afastar, por várias coisas*” (E6 - menor quintil de QARPSSA).

### Classe 3 - Integralidade no atendimento a grupos vulneráveis

Compreende 19,81% (*f* = 62 ST) do *corpus* total analisado. Constituída por palavras e radicais no intervalo entre χ^2^ = 4,19 (Hora) e χ^2^ = 36,33 (Atender). Essa classe é composta por palavras como “Atender” (χ^2^ = 36,33); “Criança” (χ^2^ = 24,77); “Querer” (χ^2^ = 24,29); “Casa” (χ^2^ = 20,86); “Intervir” (χ^2^ = 20,57); “Médico” (χ^2^ = 16,4); “Sentir” (χ^2^ = 16,4); “Deixar” (χ^2^ = 15,74) e “Ajudar” (χ^2^ = 15,54).

As falas dos profissionais refletem um compromisso com a integralidade no atendimento a grupos vulneráveis, evidenciando a empatia e o esforço coletivo. Os profissionais reconhecem que o atendimento vai além da consulta médica, abrangendo serviços como vacinação, consultas de enfermagem e assistência social. 

“*A gente se sensibiliza nesse sentido de ajudar quando é um idoso, quando é uma mãe que chega com uma reca de menino, que chega com uma, duas criança*s (...). *Teve um caso de uma família que* (...), *todos os profissionais, médico, enfermeiro e dentista, a gente fez tipo um movimento pra ajudar*” (E5 - maior quintil de QARPSSA).

“*Bom, quando eu vejo que está tendo conflitos, eu digo, a pessoa está nervosa, querendo ser atendida e acha que alguém foi ríspida ou rude com ela* (...) *eu deixo ela falar por um dia todinho, que às vezes ela só quer falar*” (E10 - menor quintil de QARPSSA).

### Classe 4 - Humanização e adaptação no atendimento

Compreende 30,67% (*f* = 96 ST) do *corpus* total analisado. Constituída por palavras e radicais no intervalo entre χ^2^ = 3,91 (Direito) e χ^2^ = 28,53 (Tentar). Essa classe é composta por palavras como “Tentar” (χ^2^ = 28,53); “Unidade” (χ^2^ = 27,15); “Saúde” (χ^2^ = 25,78); “Trabalhar” (χ^2^ = 21,83); “Sempre” (χ^2^ = 19,93); “Paciente” (χ^2^ = 18,13); “Acolher” (χ^2^ = 17,73); “População” (χ^2^ = 17,59) e “Precisar” (χ^2^ = 17,1).

Os profissionais destacam a importância de tratar os pacientes com acolhimento e humanização, adaptando a linguagem para garantir que compreendam suas condições de saúde. Além disso, ressalta-se a prática de oferecer posicionamentos empáticos aos pacientes, evitando respostas negativas que possam gerar desconforto. 

“*É, então aqui na unidade, né? A gente sempre busca tratar o paciente com acolhimento, com respeito, de um atendimento humanizado, né? Então diante de pacientes que estão nessa faixa de pobreza, né? Nós muitas vezes temos que adequar a nossa linguagem, né? Verbal, para que ele possa entender, né*” (E4 - maior quintil de QARPSSA).

“*Então, assim, a gente tenta trabalhar alguns grupos, né? Tentar fazer uma escuta qualificada, apoiar aqueles pacientes que precisam mais, dar uma maior atenção àqueles pacientes que precisam mais*” (E9 - menor quintil de QARPSSA).

**Figura 2:**
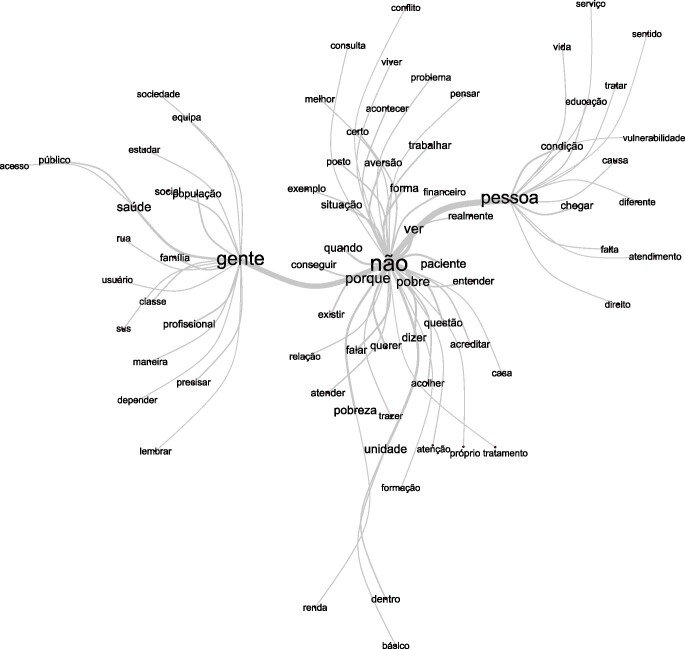
Análise de similitude.

A análise de similitude é fundamentada na teoria dos grafos em que é possível identificar as ocorrências entre as palavras e as indicações da conexão entre as palavras, auxiliando na identificação da estrutura do conteúdo de um *corpus* textual. As palavras mais próximas umas das outras no gráfico têm uma alta frequência de co-ocorrência, indicando forte relação semântica. Observa-se que a palavra “não” encontra-se no centro dos relatos, e dela ligam-se fortemente com “gente”, “pessoa” e “ver”, e a partir dessas surgem diversas outras ramificações que fundamentam todo discurso textual (Figura 2).

As palavras “não”, “pessoa” e “gente” são centrais na análise de similitude, sugerindo que elas são frequentemente usadas nas discussões sobre aporofobia. A palavra “não” aparece em conexões com “ver”, “pobre” e “paciente”, sugerindo uma negação ou dificuldade em reconhecer ou lidar com a pobreza no contexto da saúde. “Pessoa” está relacionada a palavras como “condição”, “vulnerabilidade” e “educação”, destacando questões sobre a situação de vida dos usuários. A palavra “gente” está associada a termos como “saúde”, “população” e “profissional”, indicando uma relação com a assistência à saúde das coletividades. 

## Discussão

Os relatos dos profissionais entrevistados indicaram que a aporofobia é um fenômeno presente no contexto da APS, manifestando-se de diferentes formas, desde atitudes sutis até comportamentos explícitos. A escolha proposital de participantes dos extremos da escala do QARPSSA teve como objetivo explorar diferenças de percepção sobre o tema, o que parece ter influenciado os resultados e evidenciado dualidade de atitudes.

Alguns profissionais manifestaram dificuldade em reconhecer ou lidar com a pobreza no contexto da saúde. Diante das múltiplas demandas e da complexidade do trabalho na APS ^21^, o modelo de atenção à saúde vigente impõe limitações à discussão dos processos de trabalho entre os profissionais. No cotidiano da ESF, a pressão por produtividade e as demandas assistenciais tendem a direcionar as práticas para a resolução de problemas imediatos de saúde. Essa situação compromete a identificação e o enfrentamento da aporofobia no cuidado, também dificultando a execução de dispositivos preconizados pela Política Nacional de Humanização (PNH) ^22^, como a clínica ampliada, que poderia contribuir para maior sensibilização às vulnerabilidades sociais e promover a integralidade. Dessa forma, como as condições de saúde dos usuários também estão relacionadas à sua situação de vulnerabilidade social ^23^, torna-se necessário integrar esses aspectos ao cuidado.

Esses achados indicam que preconceitos socialmente estabelecidos influenciam a percepção dos profissionais, afetando a forma como lidam com os usuários da ESF. A aporofobia compromete a qualidade do cuidado e o acesso à saúde, resultando em discriminação, desassistência e até recusa de atendimento ^3^. Populações vulneráveis, como pessoas em situação de rua ou de baixa renda, recebem menos atenção médica e são tratadas com menos respeito. Esse cenário agrava a exclusão social e os riscos à saúde, especialmente para aqueles que evitam os serviços por medo de serem maltratados ou ignorados ^10^ ^,^ ^24^.

A ESF tem demonstrado ser efetiva em melhorar diversos desfechos de saúde, incluindo a redução de desigualdades sociais em mortalidade e a promoção de cuidados de saúde equitativos ^25^. O preconceito contra populações vulneráveis no atendimento em saúde não pode ser dissociado de outros determinantes sociais importantes, como acesso precário à educação, moradia e assistência social ^26^. Dessa forma, a atuação isolada do setor saúde não é suficiente para enfrentar a aporofobia, sendo necessária a articulação de políticas públicas intersetoriais.

A exclusão social impacta a percepção dos profissionais da APS sobre os usuários, frequentemente associados à dependência de programas sociais como o Bolsa Família. Devido à falta de conhecimento sobre o funcionamento dos programas de transferência de renda, a sociedade tende a associar seus beneficiários a valores negativos, percebendo-os como preguiçosos e culpados pela própria desigualdade ^27^. Esse estigma reforça a ideia equivocada de que a pobreza é uma escolha, ignorando sua natureza estrutural. No entanto, a pobreza não define a identidade de uma pessoa ^1^. Desigualdades sociais, falta de oportunidades e políticas públicas insuficientes são fatores que perpetuam a vulnerabilidade e limitam o acesso a direitos básicos ^28^.

Outro aspecto importante é a reação de aversão que muitos profissionais manifestaram em relação à aparência física e ao estado de vulnerabilidade social dos usuários, reforçando o estereótipo de pobreza. A literatura aponta que pessoas em situação de rua são frequentemente associadas à pobreza, ao risco à saúde e à violência urbana ^29^, refletindo discriminação. Cortina ^1^ destaca que a pobreza desperta uma sensação de contaminação e desconforto social, levando à percepção de indivíduos vulneráveis como ameaças ao bem-estar e à segurança. 

Para alguns profissionais, a pobreza é vista não apenas como uma questão econômica, mas também como uma carência em termos espirituais, de comportamento e de educação, refletindo uma compreensão multidimensional da pobreza ^30^ ^,^ ^31^. Essa perspectiva pode ampliar o entendimento sobre as realidades vivenciadas pelos usuários, mas também pode reforçar preconceitos ao associar a pobreza a uma “carência” de caráter pessoal. Essa percepção leva ao questionamento das abordagens educacionais atualmente utilizadas na formação dos profissionais de saúde, que muitas vezes não incluem o combate à aporofobia e o preconceito social.

Cortina ^1^ destaca que a educação é essencial para combater a aporofobia, mas as abordagens atuais ainda são insuficientes. Profissionais de saúde têm um papel relevante na promoção da equidade, sendo fundamentais para melhorar o atendimento a populações vulneráveis ^32^. A qualidade do cuidado depende da educação permanente e da sensibilização para preconceitos próprios ^33^. A formação na área da saúde deve incluir, além de conhecimentos técnicos, a compreensão das desigualdades socioeconômicas e seus impactos na saúde. Incluir os determinantes sociais nos currículos e incentivar a autorreflexão sobre preconceitos são passos essenciais para garantir um atendimento mais justo e acessível ^34^.

A qualificação do acolhimento, diretriz central da PNH ^22^, é essencial para garantir respeito e valorização a todos os usuários nos serviços de saúde. Profissionais o reconhecem como estratégia para enfrentar vulnerabilidades socioeconômicas. Contudo, a PNH priorizou o encontro positivo entre usuários e trabalhadores, sem considerar as dimensões conflituosas e desiguais das relações, como preconceito e discriminação. Questões como aporofobia e racismo ainda são pouco discutidas no cuidado em saúde e inexistem protocolos para orientar a atuação profissional diante dessas situações na APS. No Brasil, a formação em saúde inclui comunicação, humanização e princípios de justiça social ^35^, mas o acolhimento ainda não está totalmente sistematizado nos modelos de atenção ^36^. Persistem desafios como atualizar diretrizes curriculares e formar docentes comprometidos com práticas humanizadas ^37^.

Por fim, vale destacar como as características individuais dos profissionais podem ter influenciado suas percepções sobre aporofobia. A diversidade de vínculos empregatícios pode afetar como lidam com a questão, sendo que servidores estatutários, por terem maior estabilidade, tendem a desenvolver vínculos mais fortes com os usuários. Estudo em Fortaleza mostrou que o desempenho e a satisfação no trabalho são influenciados pela forma de contratação, com melhores resultados entre estatutários ^38^. Profissionais temporários, por outro lado, enfrentam maior insegurança no emprego e menos acesso a capacitações ^39^, o que pode dificultar a construção de vínculos duradouros e comprometer a qualidade do atendimento alinhado aos princípios do SUS.

A pesquisa mostrou que profissionais negros tendem a apresentar atitudes menos preconceituosas em relação à pobreza, possivelmente por vivenciarem ou presenciarem discriminações semelhantes. Apesar de negros representarem a maioria da população brasileira, esse grupo enfrenta de forma desproporcional a pobreza, reflexos do racismo estrutural ^40^. Estima-se que 35% das pessoas negras no Brasil vivem em situação de pobreza, índice superior ao da população branca ^41^. Além disso, famílias negras pobres não estão em condição equivalente às brancas, pois enfrentam simultaneamente pobreza e racismo ^5^. Esse sistema de opressão ultrapassa atos isolados, manifestando-se de forma institucionalizada e perpetuando privilégios ^42^. Fanon ^7^ ressalta que o racismo atua como um conjunto de comportamentos socialmente estruturados que definem o lugar dos indivíduos. No campo da saúde, isso resulta na marginalização da população negra, evidenciando a reprodução constante do racismo nos serviços.

Um estudo sobre preconceito racial entre profissionais de saúde mostrou que trabalhadores negros apresentaram níveis significativamente menores de preconceito implícito em comparação aos brancos ^43^. A presença de médicos negros na força de trabalho é considerada uma estratégia para reduzir o impacto do preconceito e minimizar disparidades em saúde ^44^, reforçando a importância da diversidade racial na composição das equipes da ESF como passo inicial para promover equidade. Como destacou Nascimento ^6^, a desigualdade racial no Brasil transcende fatores econômicos e educacionais, refletindo a exclusão da população negra como sujeitos plenos de direitos.

Por fim, embora a discriminação racial não faça parte do instrumento base deste estudo, é preciso destacar a sua relevância na realidade brasileira. O Brasil, maior receptor de africanos escravizados e último país das Américas a abolir a escravidão, mantém fortes desigualdades estruturais que afetam a população afro-brasileira. Essas disparidades se expressam em diferentes âmbitos sociais, como a pobreza e o acesso precário à saúde. A teoria interseccional mostra que opressões como racismo e aporofobia não atuam de forma isolada, mas se somam e intensificam experiências de exclusão. Pessoas negras em situação de pobreza enfrentam vulnerabilidades ampliadas pela sobreposição de discriminações ^45^. Por isso, pesquisas futuras sobre aporofobia no Brasil devem considerar essa intersecção, reconhecendo que pobreza e racismo não apenas coexistem, mas se reforçam, aprofundando desigualdades e limitando o acesso a direitos básicos.

O estudo apresentou como limitação o uso do QARPSSA como *proxy* para medir a aporofobia, já que o conceito é recente e pode não ter sido plenamente capturado. Apesar disso, a adoção de abordagem mista possibilitou compreender de forma mais ampla as percepções e experiências de profissionais de saúde sobre o preconceito contra pobres. A análise das falas, embora sujeita à subjetividade, foi fortalecida pelo uso do software IraMuTeq, que reduziu vieses e permitiu validação estatística. A diversidade da amostra também ampliou a compreensão do fenômeno, trazendo múltiplas perspectivas sobre a aporofobia.

## Conclusão

A aporofobia compromete a equidade no atendimento oferecido na APS, influenciando a forma como os profissionais percebem e interagem com os usuários em situação de vulnerabilidade social. A dificuldade de alguns profissionais em reconhecer ou lidar com a pobreza no contexto da saúde, somada às situações de discriminação vivenciadas pelos usuários, revela uma barreira para se alcançar a equidade em saúde. A dificuldade em reconhecer as causas e consequências da pobreza pode estar relacionada a preconceitos, como estigmas sobre a população pobre, mas também à ausência, nos processos formativos, de discussões estruturadas sobre desigualdades sociais, vulnerabilidades e seus impactos na saúde.

É essencial que a formação dos profissionais de saúde contemple discussões sobre diferentes formas de discriminação e opressão social e determinação social da saúde, ampliando a conscientização sobre os impactos da aporofobia no cuidado à saúde. O fortalecimento do acolhimento na APS e da educação permanente com foco na humanização é uma estratégia que pode promover um cuidado mais equitativo na saúde. No entanto, o enfrentamento da aporofobia não deve se restringir ao setor saúde, sendo necessária uma articulação intersetorial com educação, assistência social e habitação para reduzir as desigualdades que perpetuam a exclusão social no acesso aos serviços de saúde e mesmo na sociedade brasileira.

## Data Availability

Os dados de pesquisa estão disponíveis mediante solicitação à autora de correspondência.
